# Vibrios from the Norwegian marine environment: Characterization of associated antibiotic resistance and virulence genes

**DOI:** 10.1002/mbo3.1093

**Published:** 2020-06-17

**Authors:** Fredrik Håkonsholm, Bjørn Tore Lunestad, Jose Roberto Aguirre Sánchez, Jaime Martinez‐Urtaza, Nachiket Prakash Marathe, Cecilie Smith Svanevik

**Affiliations:** ^1^ Institute of Marine Research Bergen Norway; ^2^ Centro de Investigación en Alimentación y Desarrollo (CIAD) Culiacán Sinaloa Mexico; ^3^ Department of Genetics and Microbiology Universitat Autònoma de Barcelona (UAB) Barcelona Spain

**Keywords:** antimicrobial resistance, marine environment, *Vibrio* spp., virulence, whole‐genome sequencing

## Abstract

A total of 116 *Vibrio* isolates comprising *V. alginolyticus* (*n* = 53), *V. metschnikovii* (*n* = 38), *V. anguillarum* (*n* = 21)*, V. antiquarius* (*n* = 2), and *V. fujianensis* (*n* = 2) were obtained from seawater, fish, or bivalve molluscs from temperate Oceanic and Polar Oceanic area around Norway. Antibiotic sensitivity testing revealed resistance or reduced susceptibility to ampicillin (74%), oxolinic acid (33%), imipenem (21%), aztreonam (19%), and tobramycin (17%). Whole‐genome sequence analysis of eighteen drug‐resistant isolates revealed the presence of genes like β‐lactamases, chloramphenicol‐acetyltransferases, and genes conferring tetracycline and quinolone resistance. The strains also carried virulence genes like *hly*A, *tlh*, *rtx*A to D and *ace*A, E and F. The genes for cholerae toxin (*ctx*), thermostable direct hemolysin (*tdh*), or zonula occludens toxin (*zot*) were not detected in any of the isolates. The present study shows low prevalence of multidrug resistance and absence of virulence genes of high global concern among environmental vibrios in Norway. However, in the light of climate change, and projected rising sea surface temperatures, even in the cold temperate areas, there is a need for frequent monitoring of resistance and virulence in vibrios to be prepared for future public health challenges.

## Graphical abstract

This study investigated the occurrence of *Vibrio* spp. in water samples, fish and marine bivalves from the temperate Oceanic and Polar Oceanic areas around Norway. Five species, *V. alginolyticus* (n = 53), *V. metschnikovii* (n = 38), *V. anguillarum* (n = 21), *V. antiquarius* (n = 2) and *V. fujianensis* (n = 2) were isolated. We examined antimicrobial susceptibility, hemolytic activity and characterized antimicrobial resistance genes and virulence genes by whole genome sequencing. This study found low prevalence of multidrug resistance, and absence of virulence genes of high global concern.

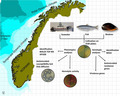

## 1 INTRODUCTION

*Vibrio* spp. have the sea and brackish water as their natural habitat and are among the most common bacteria found in surface waters worldwide (Vezzulli, Colwell, & Pruzzo, 2013). The genus includes several fish and human pathogenic species. Among these human pathogens, *V. cholerae*, *V. parahaemolyticus,* and *V. vulnificus* have been extensively studied (Baker‐Austin et al., 2018; Stavric & Buchanan, 1997).

*V. cholerae* has through history caused several pandemics and the main culprit being *V. cholerae* serotype O1/O139 encoding cholerae toxin (CTX; Islam et al., 2013). However, non‐O1/non‐O139 *V. cholerae* can also cause infections. The virulence factors of non‐O1 and non‐O139 include a heat‐stable enterotoxin, repeat in toxin (*rtx*) and El Tor hemolysin (*hlyA)* (Kumar, Peter, & Thomas, 2010). In contrast, the pathogenicity of *V. parahaemolyticus* is linked to their ability to produce a thermostable direct hemolysin (TDH), or a TDH‐related hemolysin (TRH), encoded by *tdh* and *trh* genes (Raghunath, 2015). For *V. vulnificus*, virulence is related to the production of a polysaccharide capsule and lipopolysaccharide (LPS), flagellum, hemolysin, and proteases (Roig et al., 2018). The genetic basis for human virulence is only partially known, although several studies suggest that all strains of *V. vulnificus,* regardless of their origin, may be able to cause infections in humans (Roig et al., 2018). Several other *Vibrio* spp., such as *V. alginolyticus, V. fluvialis, V. mimicus, V. metschnikovii, V. furnissii, V. hollisae,* and *V. damsela,* can occasionally cause infections in humans (Austin, 2010; Baker‐Austin et al., 2018).

*Vibrio* infections in humans typically occur as a result of ingestion of contaminated seafood, through the handling of raw seafood or by exposure of wounds to seawater during recreation (Iwamoto, Ayers, Mahon, & Swerdlow, 2010). The human pathogenic vibrios show strong seasonality and are more abundant when the water temperature exceeds 18°C and the salinity drops below 25 ‰ (Vezzulli et al., 2013). In the last decades, an increase in infections caused by *Vibrio* spp. has been reported, also in colder regions of South America and Northern Europe, including Norway, where this was previously rare (Baker‐Austin et al., 2016). One of the primary effects of climate change is increased sea surface temperatures (SSTs), and this may facilitate the spread of seawater associated diseases (EEA, 2017). The temperature is predicted to increase further in northern temperate waters (EEA, 2017), and new areas may become more favorable for the pathogenic vibrios. Several fish pathogenic vibrios have been identified and are a challenge in aquaculture. The most common *Vibrio* species infecting farmed aquatic animals are *V. parahaemolyticus*, *V. alginolyticus*, *V. harveyi*, *V. owensii*, *V. campbellii,* and *V. anguillarum* (Ina‐Salwany et al., 2019).

The role of the marine environment in the development and dissemination of antimicrobial resistance is largely unknown. Vibrios are indigenous to the sea (Banerjee & Farber, 2018), and in recent years, the occurrence of resistance genes in *Vibrio* spp. has been examined. Genes encoding resistance to β‐lactams like *penA*, *bla*_TEM‐1_ (Letchumanan, Chan, & Lee, 2015), and *bla*_VCC‐1_ (Hammerl et al., 2017; Mangat et al., 2016), chloramphenicol resistance genes, such as *floR*, *catI,* and *catII,* and several *tet* genes encoding resistance to tetracycline (Letchumanan et al., 2015), have been detected in *Vibrio* spp. Clinically important mobile resistance genes like *qnrVC* and *qnrS* have originated in *Vibrio* spp. (Fonseca, Dos Santos Freitas, Vieira, & Vicente, 2008). This makes *Vibrio* spp. a good model organism for the studying antibiotic resistance in the marine environment.

Although *V. parahaemolyticus*, *V. cholerae,* and *V. vulnificus* have previously been isolated from Norway (Bauer, Ostensvik, Florvag, Ormen, & Rorvik, 2006), there is limited knowledge on the prevalence of different *Vibrio* spp. and associated resistance and virulence markers in the Norwegian marine environment. This study aimed to examine the prevalence of different *Vibrio* spp. in the Norwegian marine environment and to characterize associated virulence and antibiotic resistance genes among these. We here present a detailed account of taxonomy, resistance, and virulence genes detected based on phenotypic culture‐based methods and whole‐genome sequence (WGS) analysis.

## 2 EXPERIMENTAL PROCEDURES

### 2.1 Sampling

**Figure 1:**
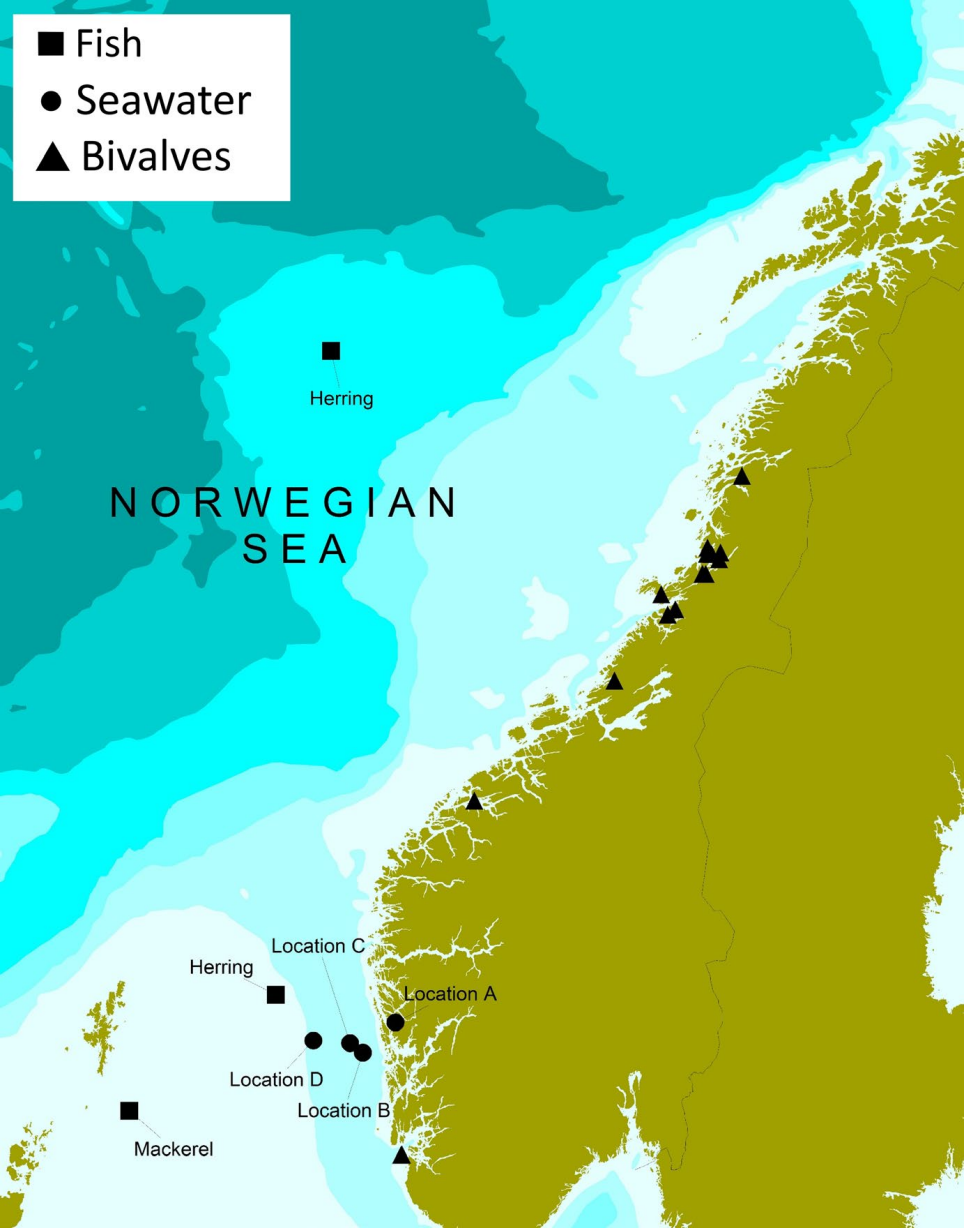
Map of Norway showing sampling locations for fish (herring and Atlantic mackerel) captured during commercial pelagic fisheries, seawater collected during the herring fisheries, and marine bivalves from harvesting areas included in the surveillance program of the Norwegian Food Safety Agency

Water samples were collected from four different locations (A–D) at the West coast of Norway (Oceanic temperate zones) at five different depths (0, 2, 5, 7, and 10 m) from each location during May 2018, comprising 20 water samples. A total of 60 fish caught in the North Sea, including 40 herring (*Clupea harrengus*) and 20 Atlantic mackerel (*Scomber scombrus*), were sampled from May to November 2018. The fish were caught by commercial fishing vessels during the catch season for the respective species. The bivalve molluscs samples were collected from 16 rearing locations along the Norwegian coast (Oceanic and Polar Oceanic zones) in November 2018 (Figure 1), through the annual surveillance program on *Escherichia coli* in bivalves by the Norwegian Food Safety Authority (NFSA). This included 14 batch samples of blue mussels (*Mytilus edulis)*, one batch sample of flat oysters (*Ostrea edulis),* and one batch sample of scallops (*Pecten maximus*), where each batch sample comprised batches of 10–15 individual bivalve molluscs. All samples were further examined at the Institute of Marine Research (IMR).

### 2.2 Isolation of *Vibrio* spp.

From each water sample, three aliquots of 100–250 ml were filtered through 0.45 µm filters (Merck Millipore, Germany) using the EZ‐fit Manifold 3‐place system (Merck Millipore, Germany) connected to a vacuum pump. Each filter was transferred to thiosulfate‐citrate‐bile‐sucrose (TCBS) agar (Oxoid, UK) plates and incubated at 37°C for 24–48 hrs. Also, an enrichment step was performed in duplicates on 500 ml water adding 50 ml concentrated (360 mg/ml) alkaline peptone water (APW) with 2% sodium chloride (NaCl). The enrichment cultures were incubated at 42 ˚C for 18 hr. After incubation, 100 µl of the enrichment cultures was streaked on TCBS agar and incubated at 37 ˚C for 24–48 hr. Typical colonies were picked from the plates and restreaked for obtaining pure cultures.

Isolation of *Vibrio* spp. from fish and bivalve molluscs followed a method based on NMKL method no. 156 (NMKL, 1997). The method takes advantage of the vibrios alkaline and halophilic properties (Vezzulli et al., 2013) and applies APW supplemented with 2% NaCl and 42°C as incubation temperature for selective enrichment of human pathogenic species (NMKL, 1997). For isolation of *Vibrio* spp., TCBS is a widely used medium. The alkaline pH (8.6), bile salts, and NaCl concentration in the agar inhibit the growth of Enterobacteriaceae and Gram‐positive organisms (Donovan & van Netten, 1995). From herring collected in June 2018, samples were taken from the skin with muscle, gills, and intestine. From each tissue type, 20 g was homogenized in 180 ml APW with 2% NaCl and APW with 2% NaCl supplemented with polymyxin B (250 IU/ml) for 30 s. using a stomacher. The homogenate was incubated at 42 ± 1°C for 18 ± 2 hrs. After incubation, 10 µl of the enrichment cultures was streaked on TCBS agar and incubated at 37 ± 1°C for 24 ± 3 hrs. From mackerel collected in September, samples were taken from the skin with mussel following the same protocol as described previously. Samples were also collected from gut content and homogenized in phosphate‐buffered saline (PBS) (Sigma‐Aldrich), and tenfold dilution series were made. From each sample, 100 µl was spread on TCBS and incubated at 37 ± 1°C for 24 ± 3 hrs. From herring collected in November, samples were collected from the skin with muscle and prepared following the same method as described previously.

From bivalve molluscs, 100 g soft tissue and intravalvular fluid from at least 10 individual bivalves were homogenized in sterile plastic bags and 20 g was transferred to new sterile bags. Enrichment followed the same protocol as for fish samples. Additionally, from the homogenate tenfold dilution series were made using peptone water (bioMerièux, France). From dilutions and undiluted samples, 100 µl was spread on TCBS and *Vibrio* ChromoSelect agar (VCS; Sigma‐Aldrich) and incubated at 37°C for 24–48 hrs followed by a selection of typical colonies.

### 2.3 Biochemical identification

Isolates were grown overnight on plate count agar (PCA) (Oxoid, UK) supplemented with 2% NaCl and characterized biochemical using the Analytical Profile Index 20E (API 20E, bioMerièux, France) following the instructions of the manufacturer. Overnight cultures were used to prepare bacterial inoculums corresponding to 0.5 McFarland in 2% sterile saline.

### 2.4 Identification by MALDI‐TOF‐MS

All isolates were grown overnight on PCA supplemented with 2% NaCl and sent to the Norwegian Veterinary Institute (NVI) in Bergen for identification by matrix‐assisted laser desorption ionization time‐of‐flight mass spectrometry (MALDI‐TOF‐MS) (Bruker, Germany). The obtained peptide mass fingerprints (PMFs) were compared to spectra in the commercial MALDI‐TOF‐MS database (MALDI Biotyper, Bruker, Germany) and to spectra in an in‐house generated database containing spectra from *Vibrio* spp. known to be associated with marine fish.

### 2.5 Whole‐genome sequencing and sequence analysis

Eighteen isolates were subjected to whole‐genome sequencing (WGS). DNA was extracted from isolates using the DNeasy Blood & Tissue kit (Qiagen, Germany). An additional lysis step was performed by resuspending the samples in 180 µl lysis buffer and incubating them at 37°C overnight. After incubation, DNA extraction was done as described by the manufacturer (Quiagen, 2006). The purity (260/280 and 260/230 ratios) and concentration in the DNA was measured using Nanodrop ND‐1000 (NanoDrop Technologies, USA) and Qubit 2.0 broad range dsDNA kit (Invitrogen, USA).

Genomic libraries were prepared using Nextera DNA Flex Tagmentation (Illumina, USA) and sequenced using the MiSeq (Illumina, USA) platform to obtain 300 bp paired‐end reads. The raw sequence data were adapter and quality trimmed using BBDuk (https://jgi.doe.gov/data‐and‐tools/bbtools/bb‐tools‐user‐guide/) and assembled using SPAdes version 3.13.1 (Bankevich et al., 2012). Assembled genomes were annotated using the NCBI Prokaryotic Genome Annotation Pipeline (Tatusova et al., 2016) and the Rapid Annotations using Subsystems Technology (RAST) server (Aziz et al., 2008). Resistance genes were detected using the Comprehensive Antibiotic Resistance Database, CARD (Jia et al., 2017), and the Resistance Gene Identifier mode. Virulence genes were detected using virulence factors database (VFDB; Liu, Zheng, Jin, Chen, & Yang, 2019).

### 2.6 Species identification of WGS

Raw forward and reverse reads in the FastQ format were uploaded to The Microbial Genomes Atlas (MiGA) (Rodriguez et al., 2018) web server in the TypeMat mode. In this mode, the sequences are trimmed, assembled, and aligned to give the closest relatives found in the MiGA Reference database.

### 2.7 Phylogenetic inference

For each *Vibrio* species (*V. metschnikovii*, *V. anguillarum,* and *V. alginolyticus*), single nucleotide polymorphisms (SNPs) were called with Harvest Suit (Treangen, Ondov, Koren, & Phillippy, 2014). Phylogenetic inference by ML was performed on the core genome with RAxML v8.1 (Stamatakis, 2014) and the GTRGAMMA model (1,000 bootstrap replicates). The resulting trees were visualized and edited using iTOL v4.3.3 (Letunic et al., 2006).

### 2.8 Antimicrobial susceptibility testing

Antimicrobial susceptibility testing of isolated *Vibrio* spp. was conducted by disk diffusion according to the Clinical and Laboratory Standards Institute (CLSI) method M42‐A (CLSI, 2006). Each isolate was tested against 18 antibiotics belonging to 10 different classes commonly used for either human administration, agriculture, veterinary medicine, or aquaculture in Norway (NORM/NORM‐VET, 2018) using. These included mecillinam (10 µg), ampicillin (10 µg), cefotaxime (5 µg), ceftazidime (10 µg), doxycycline (30 µg), tetracycline (30 µg), ciprofloxacin (5 µg), oxolinic acid (2 µg), imipenem (10 µg), meropenem (10 µg), erythromycin (15 µg), azithromycin (15 µg), sulfamethoxazole/trimethoprim (25 µg), trimethoprim (5 µg), gentamycin (10 µg), tobramycin (10 µg), florfenicol (30 µg), and aztreonam (30 µg). *V. alginolyticus*, *V. metschnikovii,* and *V. anguillarum* were incubated at 28°C. *E. coli* CCUG17620 was included as quality control in each setup. Inhibition zones were interpreted according to breakpoints for Enterobacteriaceae from CLSI method M100 (CLSI, 2017). For oxolinic acid, erythromycin and florfenicol breakpoints and epidemiological cutoff values (ECVs) for *Aeromonas salmonicda* from CLSI VET03/VET04 (CLSI, 2014) were used.

For isolates showing reduced susceptibility for imipenem, MIC values were determined following CLSI method M42‐A using MIC evaluator strips (Oxoid, UK).

### 2.9 CarbaNP test

Isolates showing reduced susceptibility to imipenem by the disk diffusion method were grown overnight on tryptic soy agar (TSA; Merck, Germany) at 37°C and examined for carbapenemase production by the CarbaNP test as described by Dortet, Poirel, Errera, and Nordmann (2014).

### 2.10 Hemolysis

*V. alginolyticus* and *V. metschnikovii* isolates were screened for hemolytic activity on TSA with 5% sheep blood (VWR, USA) or TSA with 5% human blood. Agar plates containing human blood were prepared by using TSA (Merck, Germany) as a base and supplemented with 5% EDTA blood. Isolates were cultivated on TSA and incubated at 37°C for 24 hrs.

## 3 RESULTS

### 3.1 Physical parameters and bacteria plate count

**Figure 2:**
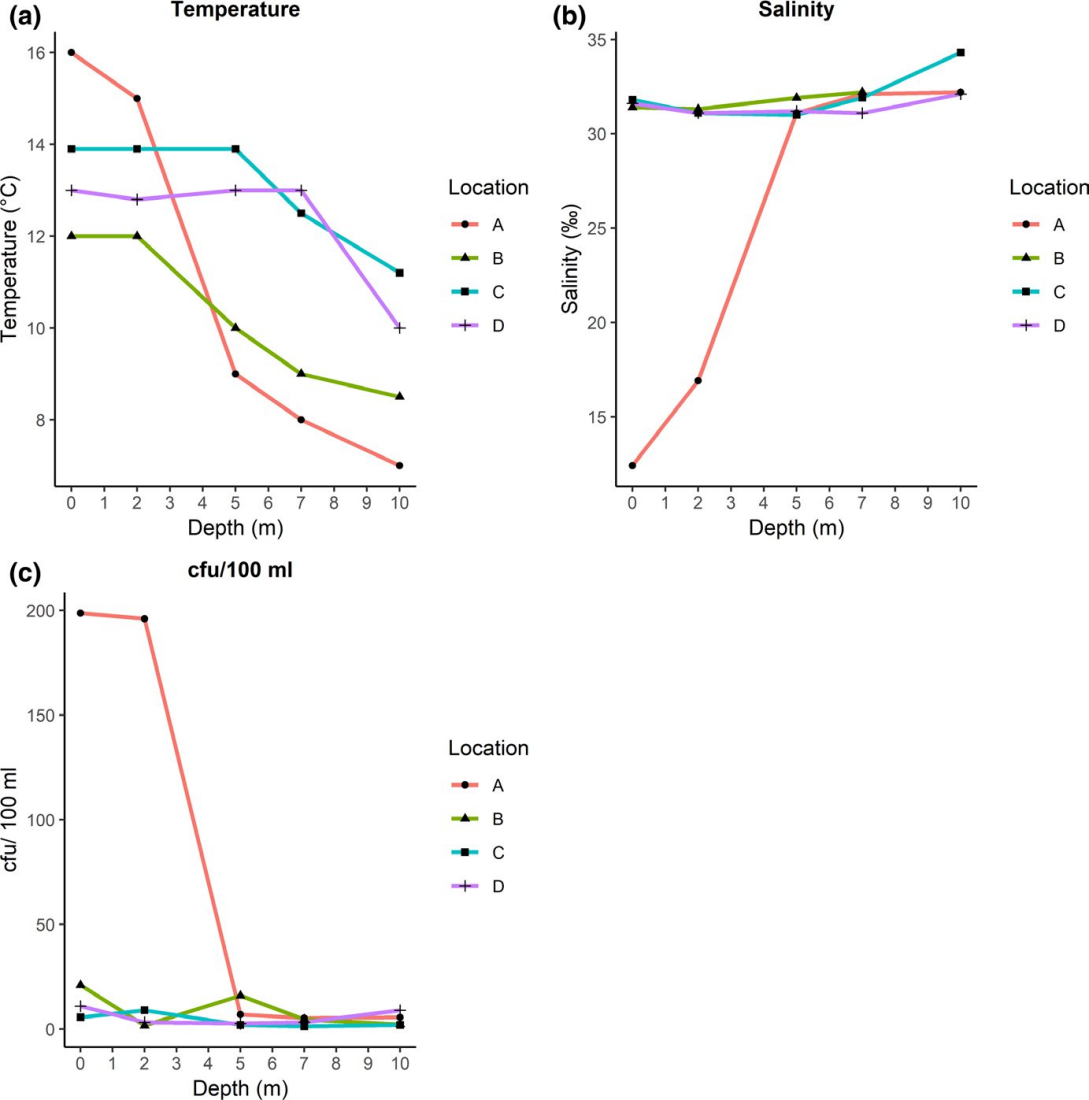
Physical parameters in seawater samples collected during herring fisheries, locations A, B, C, and D (Figure 1). (a) Measured temperature (°C). (b) Measured salinity (‰), note: missing measurement at 10 m from location B. (c) Number of colony‐forming units (cfu)/100 ml water on TCBS plates incubated at 37°C for 24–48 hrs.

The highest measured temperatures in seawater samples were seen close to the shore at location A (Figure 1), in samples collected in the surface and at 2 m depth, with temperatures of 16°C and 15°C, respectively (Figure 2a). All other samples had temperatures <15°C. In the seawater samples at location A (surface and 2 m), salinity of 12.4‰ and 16.9‰ was observed, which was the lowest of all samples (Figure 2b). The highest plate counts (cfu/100 ml) on thiosulfate‐citrate‐bile‐sucrose agar (TCBS) were observed in the samples with the highest temperature and lowest salinity (Figure 2c).

### 3.2 Prevalence and identification of *Vibrio* spp

Colonies were selected for further characterization based on morphology on TCBS agar (NMKL, 1997) and color formation on *Vibrio* ChromoSelect agar (VCS). Presumptive *Vibrio *spp. were detected in 50% of water samples, 33% of fish samples, and 31% of bivalve molluscs samples. In total, 60 isolates were recovered from water samples, 32 from fish and 24 from bivalves. Using Analytical Profile Index (API) 20E, 54 (47%) of the 116 isolates were identified as *Vibrio* spp., 49 of which were *V. alginolyticus,* three *V. cholerae,* and two *V fluvialis*. The remaining isolates were identified as members of *Aeromonas*, *Pasteurella*, *Shewanella*, and *Proteus* or yielded an “Unacceptable profile.”

One hundred and fifteen (99%) of the 116 isolates were identified as *Vibrio* spp. by matrix‐assisted laser desorption ionization time‐of‐flight mass spectrometry (MALDI‐TOF‐MS), and one isolate could not be identified. The MALDI‐TOF‐MS identified vibrios belonging to the three species *V. alginolyticus* (*n* = 53), *V. metschnikovii* (*n* = 38), and *V. anguillarum* (*n* = 24), respectively.

The Microbial Genomes Atlas (MiGA) run for 18 sequenced isolates identified seven *V. alginolyticus*, five *V. anguillarum,* two *V. metschnikovii*, two *V. antiquarius,* and two *V. fujianensis*. Incompliance between the identification by WGS MiGA and MALDI‐TOF‐MS was seen for the two isolates 1‐2(7‐a) and 11‐4(1), identified as *V. antiquarius* and *V. alginolyticus*, the two isolates 3‐2(1) and 2‐2(8), identified as *V. alginolyticus* and *V. anguillarum*, and for the one isolate 1‐1(7) identified as *V. fujianensis* and *V. anguillarum,* respectively.

Among the species considered to be opportunistic human pathogens (Austin, 2010; Baker‐Austin et al. 2018), *V. alginolyticus* was isolated from water, herring, and bivalves, while *V. metschnikovii* was isolated from herring and water samples. On the other hand, species harboring virulence genes but not known to cause human disease, like *V. antiquarius* (Dahanayake, De Silva, Hossain, Shin, & Heo, 2018; Nur et al., 2015) and *V. fujianensis* (Fang et al., 2018), were isolated from water only. *V. anguillarum*, a well‐known fish pathogen (Ina‐Salwany et al., 2019) rarely associated with serious human infection (Sinatra & Colby, 2018), was only isolated from bivalves.

**Figure A1:**
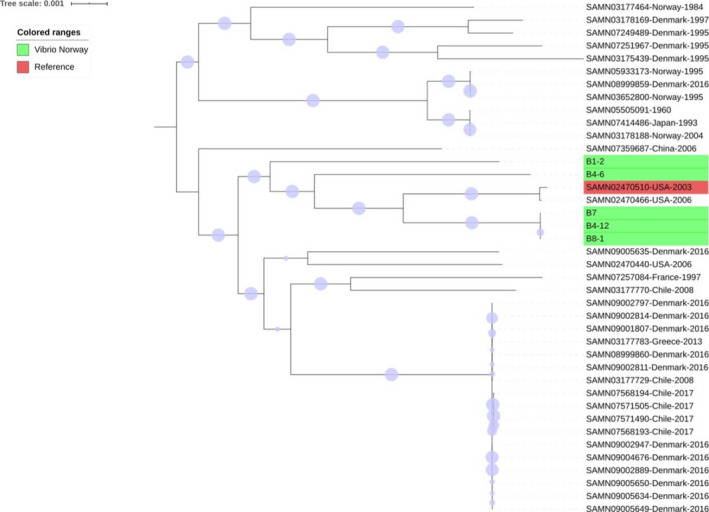
ML phylogenetic inference of *Vibrio anguillarum* strains included in this study. Genome used as reference is red shaded, while the genomes from this study are in green. Blue dots show nodes with bootstrap values above 85%

**Figure A2:**
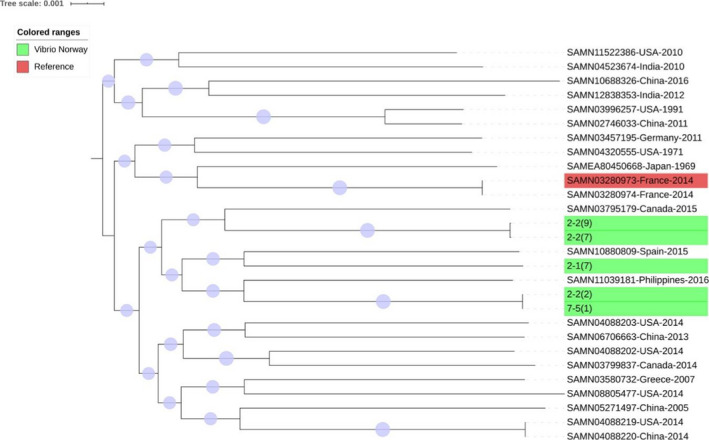
ML phylogenetic inference of *Vibrio alginolyticus* strains included in this study. Genome used as reference is red shaded, while the genomes from this study are in green. Blue dots show nodes with bootstrap values above 85%

Global mapping of the sequenced isolates of *V. alginolyticus* and *V. anguillarum* (Figures A1 and A2) showed that *Vibrio* isolates from Norway had high similarity to strains from other countries and continents, including the United States and China, indicating a global presence of these strains.

### 3.3 Hemolytic activity on blood agar

None of the 53 V*. alginolyticus* isolates displayed hemolysis on blood agar. All 38 V*. metschnikovii* isolates were hemolytic on both sheep and human blood. On sheep blood, five *V. metschnikovii* isolates were β‐hemolytic, while the remaining isolates were α‐hemolytic on both media.

### 3.4 Characterization of virulence determinants in WGS

Eighteen drug‐resistant isolates were subjected to WGS. Detailed overview of genome assembly statistics and GenBank accession numbers is presented in Table A2. Several genes related to virulence were detected in the examined genomes, including genes for mannose sensitive hemagglutinin (*msh*), adherence, type IV toxin‐coregulated pilus (*tcp*), type IV pilus (*pil*), capsular antiphagocytosis polysaccharides (*rml, vbf, cps, wec, wza, wzb, wzb*), flagellar formation genes (*che*, *fil*, *fla*, *flg*, *flh, fli, flr, mot, che*), iron uptake (*irg, vct, viu, vib, vie*), quorum sensing genes (*eps*), ESP secretion systems (*esp, gsp*), T3SS1 secretion systems, VAS effector proteins, endotoxin production, and immune evasion genes. None of the isolates carried genes for cholerae toxin production (*ctx*A or *ctx*B), thermostable direct hemolysin (*tdh*), or zonula occludens toxin (*zot*).

The most prominent virulence genes detected in this study were related to hemolysins. All *Vibrio* species examined had genes coding for the *Aeromonas*‐related hemolysin type III (Hemolysin III). The *V. cholerae* cytolysin A (*hlyA)* was detected among *V. alginolyticus*, *V. metschnikovii,* and *V. anguillarum,* whereas the thermolabile hemolysin gene (*tlh*) was present in all isolated species except *V. fujianensis*. A variety of the repeats‐in‐toxin holotoxins genes (*rtx*A to D) was detected in *V. alginolyticus*, *V. metschnikovii*, *V. anguillarum,* and *V. fujianensis*. The accessory *V. cholerae* enterotoxin genes *ace (*A, E, and F) were found in the two *V. fujianensis* isolates (Table 2).

### 3.5 Antimicrobial resistance

The phenotypic antimicrobial susceptibility testing of the 116 *Vibrio* spp. showed 74% to be resistant to ampicillin, 33% to oxolinic acid, 21% to imipenem, 19% to aztreonam, and 17% to tobramycin (Table 1). All isolates were susceptible to tetracycline, ciprofloxacin, and trimethoprim‐sulfamethoxazole, while most isolates were susceptible to third‐generation cephalosporins (98%) and aminoglycosides (83%). For the isolates showing reduced susceptibility (intermediate resistance) to imipenem, minimum inhibitory concentration (MIC) for imipenem ranged from 2 to 8 µg/ml. Detailed overview of the individual inhibition zones obtained from disk diffusion test is included in Table A1.

**Table 1:** Antibiotic sensitivity pattern among the *Vibrio* isolates

Agent	V. alginolyticus			V. metschnikovii			V. anguillarum			V. antiquarius			V. fujianensis		
	(*n* = 53)			(*n* = 38)			(*n* = 21)			(*n* = 2)			(*n* = 2)		
	S (%)	I (%)	R (%)	S (%)	I (%)	R (%)	S (%)	I (%)	R (%)	S (%)	I (%)	R (%)	S (%)	I (%)	R (%)
AMP	0	0	100	79	0	21	0	0	100	0	0	100	0	0	100
MEL	100	0	0	100	0	0	100	0	0	100	0	0	100	0	0
CTX	100	0	0	97	3	0	95	5	0	100	0	0	100	0	0
CAZ	100	0	0	97	3	0	100	0	0	100	0	0	100	0	0
TE	100	0	0	100	0	0	100	0	0	100	0	0	100	0	0
DO	100	0	0	100	0	0	100	0	0	100	0	0	100	0	0
IPM	96	4	0	100	0	0	0	0	100	100	0	0	50	50	0
MEM	100	0	0	100	0	0	100	0	0	100	0	0	100	0	0
E	98	0	2	100	0	0	71	0	29	100	0	0	100	0	0
SXT	100	0	0	100	0	0	100	0	0	100	0	0	100	0	0
W	98	2	0	100	0	0	100	0	0	50	50	0	100	0	0
OA	34	62	4	97	3	0	100	0	0	0	100	0	100	0	0
CIP	100	0	0	100	0	0	100	0	0	100	0	0	100	0	0
CN	100	0	0	82	18	0	100	0	0	100	0	0	100	0	0
TOB	100	0	0	47	39	13	100	0	0	100	0	0	100	0	0
FFC	100	0	0	100	0	0	100	0	0	100	0	0	100	0	0
AZM	100	0	0	100	0	0	100	0	0	100	0	0	100	0	0
ATM	98	2	0	100	0	0	5	86	10	100	0	0	50	50	0

Abbreviations: AMP: Ampicillin, MEL: Mecillinam, CTX: Cefotaxime, CAZ: Ceftazidime, TE: Tetracycline, DO: Doxycycline, CIP: Ciprofloxacin, OA: Oxolinic acid, IPM: Imipenem, MEM: Meropenem, E: Erythromycin, AZM: Azithromycin, SXT: Sulfamethoxazole/Trimethoprim, W: Trimethoprim, TOB: Tobramycin, CN: Gentamicin, FFC: Florfenicol, ATM: Aztreonam, S: Susceptible, I: intermediate, R: Resistant.

### 3.6 Examination of carbapenemase production

Among the 116 *Vibrio* isolates examined, resistance to imipenem was observed in all *V. anguillarum* isolates, while two *V. alginolyticus* isolates and one *V. fujianensis* isolate were intermediately susceptible to the agent. These imipenem‐resistant isolates were also resistant to ampicillin but susceptible to meropenem. All but one *V. anguillarum* isolate (B4‐12) was susceptible to cefotaxime. CarbaNP test was negative for all isolates, suggesting the absence of carbapenemase with high hydrolytic activity.

### 3.7 Genetic characterization of resistance determinants

The sequenced genomes revealed the presence of β‐lactamases like *bla*_CARB _genes in *V. alginolyticus*, *V. metschnikovii,* and *V. antiquarius* and *amp*C genes in *V. alginolyticus, V. anguillarum,* and *V. antiquarius* (Table 2). One *V. anguillarum* isolate harbored *varG* metallo‐β‐lactamase first described in *V. cholerae* (Hong‐Ting Victor et al., 2017). Genes encoding *catB*‐related o‐acetyltransferase, involved in acetylation of chloramphenicol, were detected in isolated *V. metschnikovii* and *V. anguillarum*, while genes encoding tetracycline resistance (*tet34* and *tet35*) and multidrug membrane fusion protein (*adeF*) were found in all examined sequences from *V. alginolyticus*. *V. alginolyticus* also harbored genes encoding the *qnr* family pentapeptide repeat proteins conferring reduced susceptibility against quinolones (Marathe et al., 2019).

**Table 2:** Isolation source, identification, and list of resistance and virulence genes detected in whole‐genome sequences of *Vibrio* spp. isolates

Sample	Isolate	MiGA TypeMat	p‐value	MALDI‐TOF‐MS	Score	API 20E	ID %	T‐value	Resistance genes	Virulence genes	Accession No.
Bivalve	B4‐6	*V. anguillarum*	0.004	*V. anguillarum*	2.39	*A. hydrophila/caviae/sobria*2	70.5	0.56	*bla*_ampC_, *tet(34)*	Hem III, *hyl*A, *tlh rtx*A, B, C, D	VHSL00000000
Bivalve	B7	*V. anguillarum*	0.004	*V. anguillarum*	2.48	*A. hydrophila/caviae/sobria*2	70.5	0.56	*catB*‐related, *tet(34)*	Hem III, *hyl*A, *tlh*, *rtx*A, B, C, D	VHSN00000000
Bivalve	B1−2	*V. anguillarum*	0.004	*V. anguillarum*	2.32	*A. hydrophila/caviae/sobria*1	54.2	0.36	*bla*_ampC_, varG, catB‐related, *tet(34)*	Hem III, *hyl*A, *tlh*, *rtx*A, B, C, D	VHSK00000000
Bivalve	B4−12	*V. anguillarum*	0.004	*V. anguillarum*	2.38	*A. hydrophila/caviae/sobria*2	70.5	0.56	*bla*_ampC_, catB‐related, *tet(34)*	Hem III, *hyl*A, *tlh*, *rtx*A, B, C, D	VHSM00000000
Bivalve	B8−1	*V. anguillarum*	0.004	*V. anguillarum*	2.38	*A. hydrophila/caviae/sobria*2	70.5	0.56	*bla*_ampC_, catB‐related, *tet(34)*	Hem III, *hyl*A, *tlh*	VHSO00000000
Herring	A8−1	*V. metschnikovii*	0.0094	*V. metschnikovii*	1.78	Identification not valid	‐	‐	*adeF*, *bla*_CARB_, *catB*‐related,	Hem III, *hyl*A, *tlh*	VHTC00000000
Herring	A11	*V. metschnikovii*	0.0094	*V. metschnikovii*	1.78	Unacceptable profile	‐	‐	*bla*_CARB_, *catB*‐related	Hem III*, hyl*A, *tlh*	VHSI00000000
Seawater	2‐1 (7)	*V. alginolyticus*	0.0016	*V. alginolyticus*	2.2	*V. alginolyticus*	97.8	0.74	*adeF*, *bla*_CARB_, *bla*_ampC_, *catB*‐related, *qnr*, *tet(34)*, *tet(35)*	Hem III, *tlh*	VHSR00000000
Seawater	2‐2 (2)	*V. alginolyticus*	0.0016	*V. alginolyticus*	2.09	*V. alginolyticus*	85.9	0.81	*adeF*, *bla*_CARB_, *catB*‐related, *qnr*, *tet(34)*, *tet(35)*	Hem III, *tlh*	VHSS00000000
Seawater	2‐2 (7)	*V. alginolyticus*	0.004	*V. alginolyticus*	2.21	*V. alginolyticus*	85.9	0.81	*adeF*, *bla*_CARB_, *bla*_ampC_, *qnr*, *catB*‐related, *tet(34)*, *tet(35)*	Hem III, *tlh*	VHST00000000
Seawater	2‐2 (9)	*V. alginolyticus*	0.0016	*V. alginolyticus*	2.19	*V. alginolyticus*	97.8	0.74	*adeF*, *bla*_CARB_, *bla*_ampC_, *qnr*, *catB*‐related, *tet(34)*, *tet(35)*	Hem III, *tlh*	VHSV00000000
Seawater	7‐5 (1‐a)	*V. alginolyticus*	0.0016	*V. alginolyticus*	2.11	*V. alginolyticus*	85.9	0.81	*adeF*, *bla*_CARB_, *qnr*, *catB*‐related, *tet(34)*, *tet(35)*	Hem III, *tlh*	VHSX00000000
Seawater	3‐2 (1)	*V. alginolyticus*	0.0016	*V. anguillarum*	2.37	*A. hydrophila/caviae/sobria*2	69.8	0.28	*catB*‐related, *tet(34)*	Hem III, *hyl*A, *tlh rtx*A, B, C, D	VHSW00000000
Seawater	2‐2 (8)	*V. alginolyticus*	0.0016	*V. anguillarum*	2.37	Unacceptable profile	‐	‐		Hem III, *hyl*A, *tlh rtx*A, B, C, D	VHSU00000000
Seawater	1‐2 (7‐a)	*V. antiquarius*	0.0048	*V. alginolyticus*	2.21	*V. alginolyticus*	97.8	0.74	*adeF*, *bla*_CARB_, *catB*‐related, *qnr*, *tet(34)*, *tet(35)*	Hem III, *tlh*	VHSQ00000000
Seawater	11‐4 (1)	*V. antiquarius*	0.004	*V. alginolyticus*	2.13	*V. alginolyticus*	85.9	0.81	*adeF*, *bla*_CARB_, *bla*_ampC_, *catB*‐related, *qnr*, *tet(34)*, *tet(35)*	Hem III, *tlh*	VHSY00000000
Seawater	1‐1 (7)	*V. fujianensis*	0.0008	*V. anguillarum*	2.32	*A. hydrophila/caviae/sobria*1	37.3	0.33	*bla*_ampC_, *tet(34)*	Hem III, *ace*E, *ace*F, *rtx*A	VHSP00000000
Seawater	12‐2(3a)	*V. fujianensis*	0.0008	Id. not possible	‐	Unacceptable profile	‐	‐		Hem III, *ace*E, *ace*F	VMQP00000000

Abbreviations: CARB: Carbenicillin‐hydrolyzation, *catB‐*related o‐acetyltransferase involved in chloramphenicol resistance, *qnr* family pentapeptide repeat protein involved in quinolone target protection, *ade*F is the membrane fusion protein of the multidrug efflux complex *ade*FGH, and *tet(34)* as well as *tet(35)* conferring resistance to tetracyclines. *hly*A: *V. cholerae* cytolysin A, *tlh*: Thermolabile hemolysin, Hem III: *Aeromonas*‐related hemolysin type III, *rtx*A to D repeats‐in‐toxin holotoxins, *ace*A, E and F accessory cholerae enterotoxin genes.

## 4 DISCUSSION

To the best of our knowledge, this study is the most comprehensive assessment of vibrios from the Norwegian marine environment describing the prevalence of *Vibrio* spp. in Norwegian pelagic fish, bivalves, and seawater, and their characteristics concerning antimicrobial resistance and virulence.

### 4.1 Prevalence of *Vibrio* spp. in the Norwegian marine environment

The highest plate count of aquatic bacteria was observed in the water samples collected closest to the shore, where the measured temperature was highest and the salinity lowest (Location A). A total of 67% of isolated *V. alginolyticus* were isolated from these samples, where the temperature was measured to above 15°C and the salinity to ≤25 ‰, close to the preferred conditions for vibrios (Vezzulli et al., 2013; Vezzulli, Pezzati, Brettar, Höfle, & Pruzzo, 2015). *V. alginolyticus* is usually the dominating species in *Vibrio* communities (Fu et al., 2016), and our results are in accordance with this study. From fish samples, *V. metschnikovii* was the dominating species, while *V. anguillarum* was the species most frequently isolated from bivalves. Of the vibrios isolated from water samples, only four *V. alginolyticus* isolates and one *V. metschnikovii* isolate were recovered from enrichment cultures, indicating a suboptimal enrichment method for water samples. *V. vulnificus*, *V. cholerae,* and *V. parahaemolyticus* all grow at 42°C (NMKL, 1997), and hence, 42°C is used as enrichment temperature for these species. Although such a high incubation temperature may affect the recovery of stressed cells (Huq et al., 2012), Bauer et al. (2006) showed that there was no difference in isolation rate of *V. parahaemolyticus* with enrichment at 37°C and 41.5°C for isolation of *Vibrio* spp. from bivalves.

In a previous study, three major pathogenic *Vibrio* spp. (*V. vulnificus*, *V. parahaemolyticus,* and *V. cholerae*) were isolated from the Norwegian marine environment (Bauer et al., 2006). In summer of 2018, several *Vibrio* infections were reported after bathing along the Southeast coast of Norway (Naseer et al., 2019). However, none of these species was isolated in this study. Most of the samples that were obtained for this study were from the west coast of Norway, where the seas are influenced by the North and Atlantic Ocean. As a result, the sea temperature in these areas is normally low and the salinity is high. It is well known that the human pathogenic vibrios are most abundant at elevated sea temperatures, >18°C, and at lower salinity levels, <25‰ (Vezzulli et al., 2013). This may explain the absence of the major human pathogenic *Vibrio* spp. in this study. The risk of increased numbers of vibrios due to elevated temperatures is greater in the east coast of Norway and closer toward the Baltic sea (Escobar et al., 2015) where the seas are less affected by the open oceans.

### 4.2 Antimicrobial susceptibility

For the treatment of infections caused by non‐cholerae *Vibrio* spp., tetracyclines, fluoroquinolones, and third‐generation cephalosporins are among the recommended agents (Elmahdi, DaSilva, & Parveen, 2016; Wong, Brown, Luscombe, Wong, & Mendis, 2015). Resistance to these agents has been reported within the genus (Hernández‐Robles et al., 2016; Lee, Ab Mutalib, Law, Wong, & Letchumanan, 2018; Letchumanan et al., 2015). All *Vibrio* spp. isolated during this study were phenotypically susceptible to tetracycline, doxycycline, meropenem, sulfamethoxazole/trimethoprim, ciprofloxacin, florfenicol, mecillinam, and azithromycin.

Consistent with previous reports, a high prevalence of resistance to ampicillin was observed in all *Vibrio* spp. isolates in our study (Banerjee & Farber, 2018; Chiou, Li, & Chen, 2015; Hernández‐Robles et al., 2016; Li et al., 1999; Pan et al., 2013), and this resistance is usually due to the presence of a *bla*_CARB_ gene (Chiou et al., 2015; Li et al., 2016). The *bla*_CARB_ *‐*like genes have been found in *V. cholerae* predating the introduction of penicillins (Dorman et al., 2019). In this study, the *bla*_CARB_ genes were detected in *V. alginolyticus, V. metschnikovii,* and *V. antiquarius*. Genes encoding *amp*C β‐lactamase were found in *V. alginolyticus, V. anguillarum,* and *V. fujianensis*, which is conflicting to the results from phenotypic susceptibility testing as all these isolates were susceptible to cephalosporins. This may indicate that the breakpoints used in this study are insufficient for detection of these enzymes by a phenotypic method. This also highlights the need for establishing breakpoints for environmental *Vibrio* species. However, differences between phenotype and genotype may also be caused by a variable expression of genes in tested isolates (Sundsfjord et al., 2004).

A study on the antimicrobial susceptibility of environmental *V. alginolyticus* isolated from oysters in Mexico reported a high prevalence of resistance to tetracycline (Hernández‐Robles et al., 2016). Although all isolates in our study were susceptible to both tetracycline and doxycycline, the tetracycline enzymatic inactivation gene *tet*34 (Akinbowale, Peng, & Barton, 2007) and efflux encoding gene *tet*35 were frequently detected within the examined genomes in the current study.

Resistance to oxolinic acid has been reported in *V. alginolyticus* (Scarano et al., 2014), and the prevalence of reduced susceptibility was quite high in this study. All examined isolates of *V. alginolyticus* carried the *qnr* gene. It has been suggested that the marine bacteria may constitute the origin of plasmid‐mediated quinolone resistance (PMQR) genes (Poirel, Cattoir, & Nordmann, 2012) and vibrios might act as a reservoir for these genes (Poirel, Liard, Rodriguez‐Martinez, & Nordmann, 2005).

Genes encoding chloramphenicol resistance are frequently found in examined *Vibrio* spp. (Letchumanan et al., 2015), and in the current study, *V. metschnikovii* and *V. anguillarum* harbored the *catB*‐like acetyltransferase able to inactivate chloramphenicol. This gene, however, does not give resistance to florfenicol (Schwarz, Kehrenberg, Doublet, & Cloeckaert, 2004), which was the only amphenicol tested in our study.

Reduced susceptibility to aminoglycoside has been reported in clinical isolates of *V. metschnikovii* (Macarena Pariente, Elena Escribano, Liria, & S. & María Dolores Crespo, S., 2008; Wallet, Tachon, Nseir, Courcol, & Roussel‐Delvallez, 2005). This was observed quite frequently in our study; however, none of the acetyltransferases known to confer resistance to this class of agents was detected in the isolates subjected to WGS. Several efflux pumps, including members of the RND, MATE, and ABC family, were found in the isolates, but these have not been investigated in detail in our study. Pumps within these families are involved in the efflux of several classes of antibiotics, including aminoglycosides (Andersen et al., 2015; Garneau‐Tsodikova & Labby, 2016; Krause, Serio, Kane, & Connolly, 2016). Phenotypic susceptibility testing and determination of MIC indicated the presence of resistance to imipenem in all isolated *V. anguillarum*. Furthermore, two *V. alginolyticus* isolates and one *V. fujianensis* isolate were intermediately resistant to imipenem. However, none of these isolates produced positive results in the carbaNP test indicating another resistance mechanism than the production of a carbapenemase, or an imipenem hydrolyzing enzyme with a slow turnover rate (Verma et al., 2011). The observed resistance is likely caused by an alteration in porins, the presence of low‐affinity penicillin‐binding proteins or overexpression of *amp*C (El Amin et al., 2001; Nordmann, Dortet, & Poirel, 2012; Zapun, Contreras‐Martel, & Vernet, 2008). One *V. anguillarum* isolate carried gene encoding a VarG subclass B1‐like lactamase, an enzyme with the ability to hydrolyze most β‐lactam antibiotic, including cephalosporins and carbapenems (Lin et al., 2017). This isolate was, however, susceptible to both meropenem and cephalosporins.

### 4.3 Virulence

Members of the genus *Vibrio* are known to possess a range of virulence factors connected to adherence (ACF, IlpA, MAM7, MSHA pili, OmpU, TCP, VpadF), pili production, motility by flagella, regulation (AI‐2, CAI‐1), iron uptake, secretion system (T3SS1, T3SS2, T6SS), or toxin production (Ace, CT, MARTX, TDH, TRH, VCC, Zot, RTX), often arranged in pathogenicity cassettes and islands (VPI, VPI‐2) (Pérez‐Reytor, Jaña, Pavez, Navarrete, & García, 2018).

The lack of cholerae toxin (*ctx*A or *ctx*B) production, thermostable direct hemolysin (*tdh*), or zonula occludens toxin (*zot*) indicates a low level of virulence among the examined isolates. The most common virulence genes among the isolates included in this study were the *Aeromonas*‐related hemolysin type III (Hemolysin III) (Goncalves Pessoa et al., 2019). The *V. cholerae* cytolysin A gene (*hlyA)* was found among *V. alginolyticus*, *V. metschnikovii,* and *V. anguillarum,* whereas the thermolabile hemolysin gene (*tlh*) was present in all species except *V. fujianensis*. Different repeats‐in‐toxin holotoxins (*rtx*A to D) were detected in *V. alginolyticus*, *V. metschnikovii*, *V. anguillarum,* and *V. fujianensis*.

The hemolysins produced by *V. metschnikovii* is known to lyse cells from several animals, including humans, sheep, and horse (Miyake, Honda, & Miwatani, 1988). All the *V. metschnikovii* isolates were α‐hemolytic on tryptic soy agar (TSA) with 5% human blood and on TSA with sheep blood, except five isolates that were β‐hemolytic on TSA with sheep blood. The results indicate that sheep erythrocytes are more susceptible to these hemolysins, even though a previous study showed the opposite, where human cells were more susceptible to the hemolysins produced by *V. metschnikovii* (Matté et al., 2007).

RTX is a pore‐forming toxin found in several pathogenic Gram‐negative bacteria (Lee, Choi, & Kim, 2008), while HlyA, also known as *V. cholerae* cytolysin (VCC), is a hemolysin and cytolysin with activity against a range of eukaryotic cells (Ruenchit, Reamtong, Siripanichgon, Chaicumpa, & Diraphat, 2017) and is found in both *V. cholerae* O1 and non‐O1/non‐O139. The cytotoxic activity has previously been described in *V. metschnikovii* isolated from a leg wound (Linde et al., 2004). Even though *V. metschnikovii* have caused infections in humans, it is poorly described with regard to virulence factors, and the presence of these genes may indicate a pathogenic potential.

Horizontal gene transfer can mediate transfer not only antibiotic resistance genes but also virulence factors. *V. cholerae* virulence encoding genes, for example, zonula occludens toxin (*zot*), are encoded by prophages, and it has been suggested that the transfer of *zot* encoding phages occurs frequently in the *Vibrio* community (Castillo et al., 2018). Similarly, fragments of *V. cholerae* pathogenicity islands have been detected in *V. alginolyticus*, *V. anguillarum,* and *V. metschnikovii*, indicating that important virulence genes can be present in environmental *Vibrio* spp. (Gennari, Ghidini, Caburlotto, & Lleo, 2012).

### 4.4 Species identification

Identification and discrimination of closely related *Vibrio* spp. can be difficult (Bauer & Rørvik, 2007; Cano‐Gomez, Høj, Owens, Baillie, & Andreakis, 2015; Dieckmann, Strauch, & Alter, 2010; Moreno, Romero, & Espejo, 2002). In this study, several methods for identification of the isolates were applied. The API20E biochemical method was able to identify 47% of the isolates to the genus *Vibrio*. The API20E has a bias toward clinically relevant species (Viña‐Feas, Lozano‐Leon, de Novoa, Garcia‐Martin, & Martinez‐Urtaza, 2006) and does not include as many options for identification of environmental species. A previous study showed that this system was able to correctly identify 63.9% of the *Vibrio *spp. included in the database and performed best on the identification of *V. alginolyticus* and *V. parahaemolyticus* (O'Hara, Sowers, Bopp, Duda, & Strockbine, 2003). MALDI‐TOF‐MS is primarily designed for clinical use, and thus, the library mainly contains clinically relevant species (Santos, Hildenbrand, & Schug, 2016). By applying the Bruker standard library and an external generated library consisting of marine bacteria, MALDI‐TOF‐MS determined 99% of the 116 isolates to one of the three species of *Vibrio*. Although MALDI‐TOF‐MS can differentiate between closely related *Vibrio* spp. (Eddabra, Prévost, & Scheftel, 2012), the performance of this method is dependent on the strain catalogue in the reference library. For isolates identified by MiGA, a discrepancy with MALDI‐TOF‐MS was seen for five isolates. MiGA is based on average nucleotide identity (ANI) (Rodriguez et al., 2018), a method where WGS data are used to calculate an average similarity between homologues genomic regions shared between two genomes (Kim, Oh, Park, & Chun, 2014). MiGA can discriminate between closely related species (Rodriguez et al., 2018) and the reference database includes a large number of genomes, including the *Vibrio* spp. proposed by MALDI‐TOF‐MS (http://microbial‐genomes.org/projects/20). Hence, the results from identification by MiGA should be considered most reliable.

## 5 CONCLUSION

To the best of our knowledge, this study presents the most comprehensive assessment of vibrios from the Norwegian marine environment, where potentially human pathogenic species like *V. alginolyticus* and *V. metschnikovii* were detected. Although the low frequency of multidrug‐resistant isolates was observed, several clinically important resistance genes were detected in the *Vibrio* spp. isolates. These environmental vibrios could act as a reservoir of resistance genes in the marine environment.

## 6 ETHICS STATEMENT

None required.

## CONFLICT OF INTERESTS

None declared.

## AUTHOR CONTRIBUTION

**Fredrik Håkonsholm:** Conceptualization (equal); Data curation (equal); Formal analysis (equal); Investigation (equal); Methodology (equal); Project administration (equal); Software (equal); Visualization (equal); Writing‐original draft (equal). **Bjørn‐Tore Lunestad:** Conceptualization (equal); Data curation (equal); Project administration (equal); Resources (equal); Writing‐review & editing (equal). **Jose Roberto Aguirre‐Sanchez:** Software (equal); Writing‐review & editing (equal). **Jaime Martinez‐Urtaza:** Software (equal); Writing‐review & editing (equal). **Nachiket P Marathe:** Data curation (equal); Investigation (equal); Resources (equal); Validation (equal); Writing‐review & editing (equal). **Cecilie Smith Svanevik:** Conceptualization (equal); Data curation (equal); Methodology (equal); Project administration (equal); Supervision (equal); Validation (equal); Writing‐review & editing (equal).

## Data Availability

The genomes sequences have been deposited to GenBank: https://www.ncbi.nlm.nih.gov/nuccore under the following accession numbers: VHSL00000000, VHSN00000000, VHSK00000000, VHSM00000000, VHSO00000000, VHTC00000000, VHSI00000000, VHSR00000000, VHSS00000000, VHST00000000, VHSV00000000, VHSX00000000, VHSW00000000, VHSU00000000, VHSQ00000000, VHSY00000000, VHSP00000000, VMQP00000000.

## Appendix 1

**Table A1:** Measured inhibition zones (mm) from antimicrobial susceptibility testing of isolated *Vibrio* spp. by disk diffusion

*V. alginolyticus*	Antibacterial agent, Inhibition zone (mm)																	
	AMP	MEL	CTX	CAZ	TE	DO	CIP	OA	IPM	MEM	E	AZM	SXT	W	TOB	CN	FFC	ATM
1‐1 (4)	6	32	33	31	26	31	34	32	35	36	16/20	21	31	25	22	23	36	37
1‐1 (4‐a)	6	35	30	29	29	29	35	33	39	37	16/22	20	30	22	19	20	36	31
1‐1 (8)	6	35	32	32	28/33	31	38	35	37	42	18	21	30	22	17	19	34	31
1‐2 (7)	6	36	32	34	30	29	32	30	40	42	29	24	27	17	20	22	38	29
1‐3 (1)	6	36	32	34	31	30	32	32	42	44	21	27	31	26	21	22	38	27
1‐3 (1‐a)	6	35	30	32	29	30	34	34	41	42	21	28	32	26	21	22	38	27
1‐3 (2‐a)	6	31	28	31	30	29	32	32	35	39	21	25	34	28	21	24	39	31
1‐3 (3)	6	32	29	30	30	32	32	28	41	43	20	26	33	28	20	22	37	28
1‐3 (4)	6	34	30	31	28	29	28	28	36	40	19	24	24	16	19	20	33	25
1‐3 (4‐a)	6	34	32	34	32	30	30	29	35	41	19	25	27	16	18	20	35	28
1‐3 (4‐b)	6	31	27	29	30	29	29	28	34	37	20	24	29	25	18	19	34	25
1‐3 (5)	6	34	31	32	32	30	29	29	36	39	18	22	28	16	20	22	38	28
1‐3 (6)	6	34	33	33	32	31	31	28	39	41	22	23	32	22	21	22	37	32
1‐3 (6‐a)	6	33	34	28	30	29	30	28	38	39	20	24	29	23	19	21	36	30
1‐3 (7)	6	33	32	34	31	30	29	27	41	39	18/22	22	30	24	19	21	34	32
1‐3 (10)	6	33	27	29	29	30	30	29	37	40	20	26	33	29	18	20	36	31
2‐1 (2)	6	34	28	29	30	32	28	28	37	41	19	23	33	19	20	21	37	27
2‐1 (5)	6	36	32	34	30	30	29	26	36	36	18	24	30	19	19	20	38	30
2‐1 (6)	6	23	23	21	29	28	25	24	30	32	16	20	31	26	17	18	30	19
2‐1 (6‐a)	6	30	27	25	30	31	24	25	30	32	16	18	27	26	17	18	30	20
2‐1 (7)	6	30	23	23	29	27	24	25	31	33	16	18	28	26	17	19	31	20
2‐1 (7‐a)	6	34	30	29	29	28	28	25	34	36	18	25	31	19	19	21	37	24
2‐1 (9)	6	37	32	33	28	29	32	28	39	40	20	21	28	23	20	22	35	31
2‐2 (2)	6	36	24/33	26/34	29/34	31/38	27	27	36	36	15/21	19/25	24/28	23	18	20	29/42	22/29
2‐2 (2‐a)	6	32	29	28	28	30	35	32	37	39	19	24	32	24	19	20	35	27
2‐2 (3)	6	33	31	32	29	31	36	32	37	38	20	25	30	23	19	19	36	27
2‐2 (3‐a)	6	32	29	30	27	29	34	31	36	37	19	24	32	24	18	20	34	27
2‐2 (7)	6	33	32	30	29	30	36	35	40	39	21	25	32	25	18	20	35	27
2‐2 (8)	6	24	28	23	32	28	37	35	20	29	13	20	35	25	19	21	38	26
2‐2 (9)	6	33	31	28	28	27	27	24	‐	37	19	25	28	14	16	17	37	28
2‐3 (1)	6	29	27	27	26	26	33	33	36	35	29	24	27	21	17	18	32	23
2‐3 (5)	6	30	27	26	28	27	28	29	33	35	18/19	23	36	32	18	19	37	26
2‐3 (5‐a)	6	34	30	31	29/35	28/33	28	27	36	37	18/22	19/21	31	22	18	19	35	27
2‐3 (6)	6	37	31	32	31	32	28	27	40	42	22	25	29	21	18	19	33	32
2‐3 (6‐a)	6	37	33	34	33	32	29	27	40	40	22	25	30	22	19	20	35	31
2‐3 (9)	6	30	27	27	29	29	30	26	33	35	19	21/30	33	30	18	19	36	25
2‐3 (9‐a)	6	31	28	29	26/30	28	33	31	37	38	20	24	32	25	19	20	36	27
3‐1 (1)	6	37	32	33	31/36	31	29	28	38	42	21	26/36	33	24	19	22	38	32
3‐1 (1‐a)	6	37	32	33	29/36	28/33	27	27	36	37	16/22	20/30	33	24	21	22	35	33
3‐2 (1)	6	26	28	24	31	30	36	35	20	31	16	22	33	28	20	21	37	22
4‐1 (2)	6	38	34	33	33	31	27	27	37	38	18/22	19	29	19	18	19	35	29
4‐2 (2)	6	37	36	37	33	32	30	29	39	37	20	24	32	25	20	22	36	31
4‐3 (1)	6	30	28	27	29	28	26	27	32	33	19	21	30	29	16	18	35	24
4‐3 (2)	6	35	30	32	30	31	28	27	37	39	21	25	29	23	19	20	34	29
4‐5 (1)	6	32	27	29	28	29	26	27	34	34	18	23	31	26	18	21	38	37
4‐5 (1‐a)	6	32	28	29	30	31	29	28	40	42	21	25	32	27	21	22/25	38	24
4‐5 (2)	6	33	29	30	29	31	27	26	35	38	19	24	31	25	20	21	36	24
7‐5 (1‐a)	6	32	28	28	27	29	34	36	30	34	20	22/30	29	18	17	19	33	25
8‐1 (1)	6	31	29	30	28	30	31	32	39	41	21	24	30	21	19	19	34	26
8‐1 (1‐a)	6	31	29	28	29	30	27	28	36	38	21	23	30	20	18	19	34	25
B9‐1	6	32	31	30	31	29	31	29	35	36	20	23	29	23	20	20	35	26/38
B9‐2	6	30	27	27	29	26	30	29	33	34	18	23	28	25	19	20	35	25
B9‐3	6	33	28	28	29	29	31	30	35	37	20	24	28	24	19	19	34	24
***V. metschnikovii***																		
5‐1 (4)	31	36	27	22	26	28	36	33	39	42	21/26	28	33	29	13	16	34	25
5‐2 (1)	30	35	25	20	24	26	36	34	40	41	20	26	35	33	15	16	36	27
5‐2 (2)	31	36	27	23	27	28	35	31	37	40	20/23	25	31	28	14	16	34	24
5‐2 (3)	31	37	31	23	29	30	35	33	40	42	21	25	33	30	14	15	35	25
6‐2 (1)	29	35	26	21	28	27	40	35	41	44	23	27	33	30	14	15	34	22
7‐5 (1)	32	36	32	25	28	32	36	30	36	40	21	24	30	27	14	14	33	23
A21	6	40	32	24	28	28	36	34	36	38	18	26	34	30	13	15	32	26
A2‐2	8	38	30	26	32	30	40	40	42	44	22	26	32	28	26	17	34	28
A6	32	38	28	22	27	28	38	34	40	46	22	26	32	29	15	16	34	23
A7	30	38	28	22	28	26	40	32	42	44	20/26	26/32	34	32	13	14	36	27
A81	8	37	30	22	30	28	36	34	37	40	21	24	32	30	13	15	36	25
A82	9	36	29	26	30	30	42	42	44	44	22	26	36	32	15	16	38	28
A9	32	38	28	24	27	29	42	40	42	44	22	26	32	29	13	15	38	24
A10	32	38	30	26	31	32	41	33	41	44	20	23	29	27	11	13	32	22
A11	11	36	27	23	30	30	38	37	40	42	20	23	29	26	12	14	33	23
A12	31	36	28	22	27	28	38	36	44	44	22	24	36	30	13	15	34	27
A15	31	36	26	22	28	27	36	30	38	40	19	21	30	27	11	13	33	23
A17	29	35	26	21	27	26	33	29	37	40	19	22	31	28	12	13	34	23
TA 4‐1	8	40	38	27	30	28	42	34	43	41	21	26/28	36	32	16	18	36	34
TA 4‐2	27	40	42	44	40	29	42	36	44	43	26	28	38	35	16	18	40	36
TA 5	18	40	36	35	31	32	40	35	42	44	19	22	32	29	15	18	35	28
TA 13	30	37	30	24	26	28	38	36	46	45	28	28	34	30	15	17	36	27
TA 16	30	36	28	22	30	28	42	40	44	46	21	29	38	32	18	17	38	31
TS2	32	37	28	24	29	27	39	33	40	44	20	24	30	28	12	13	33	23
TS 4	31	38	30	22	26	26	40	36	44	45	21	25	37	35	15	15	39	30
TS 6	30	37	31	24	30	29	41	39	43	46	22	28	32	29	13	15	37	22
GA 5	27	38	27	22	27	27	36	32	38	39	23	24	33	31	13	15	35	27
GA 9	30	35	29	22	28	27	34	35	39	40	19	25	33	28	13	15	37	25
GA 10	32	38	33	26	30	32	37	33	40	43	19/24	25	32	29	‐	15	34	24
GA 13	30	39	32	24	26	27	34	33	36	41	20/27	22/25	32	29	14	15	33	25
GA 14	32	38	32	24	30	30	36	34	37	40	23	25/34	33	30	17	18	33	25
GA 16	6	37	30	26	28	26	41	40	44	45	24	25	36	34	15	17	37	30
GA 20	32	40	36	26	32	31	34	33	37	42	20/27	24/32	35	31	15	16	33	26
GS 15	31	40	29	23	28	26	34	35	37	40	20/28	22/33	37	34	19	21	31	29
A12 (T.2)	12	39	30	26	29	30	39	36	38	41	23	28	34	29	15	16	35	26
A16 (T.2)	32	40	35	28	30	28	36	32	36	43	20/28	24/33	32	31	15	15	39	25
A17 (T.2)	32	40	34	26	29	30	41	37	36	38	24	29	32	29	14	18	33	24
T7 u.f (T.2)	40	41	37	29	34	32	37	38	40	44	26	27	34	31	16	18	38	26
***V. anguillarum***																		
B1‐2	6	25	27	23	33	31	37	31	18	27	13	20	32	26	20	20	35	19
B1‐4	6	24	26	23	33	32	37	34	17	29	12	22	40	26	19	21	38	21
B4‐1	6	26	28	23	32	30	39	35	18	28	12	18	35	26	18	20	36	18
B4‐3	6	24	28	22	32	30	42	39	19	30	16	21	33	26	19	20	35	18
B4‐4	6	25	27	23	32	31	41	36	18	29	14	16	35	27	19	20	38	20
B4‐5	6	26	27	24	32	30	41	37	19	30	14	20	33	26	19	20	36	20
B4‐6	6	24	27	23	31	29	39	35	18	28	13	19	32	24	18	19	35	17
B4‐7	6	25	28	23	32	31	42	37	17	29	14	21	35	25	19	20	38	19
B4‐8	6	25	27	22	31	31	41	36	19	30	16	20	32	25	19	19	38	18
B4‐9	6	26	29	23	36	32	40	36	18	29	13	20	31	24	19	20	35	17
B4‐10	6	25	27	23	33	30	39	35	19	29	14	20	30	23	19	19	35	18
B4‐11	6	25	29	24	32	30	40	36	19	29	13	20	31	24	18	19	37	18
B4‐12	6	27	25	22	34	32	42	38	19	30	16	21	33	25	19	20	35	18
B4‐13	6	27	28	24	36	34	40	36	18	29	15	21	36	27	20	21	37	20
B4‐14	6	25	27	23	32	31	38	36	18	29	14	20	33	25	19	20	37	18
B4‐15	6	27	29	25	34	31	41	36	18	30	16	21	34	25	20	21	36	18
B4‐16	6	27	29	25	36	34	43	38	19	31	15	23	36	28	20	21	37	20
B4	6	25	28	24	32	30	43	37	17	30	15	22	33	24	19	20	36	19
B7	6	24	27	24	32	31	42	37	18	29	15	23	34	24	18	19	36	18
B8‐1	6	27	28	24	34	32	42	38	18	30	15	22	34	25	19	20	39	19
B8‐2	6	25	29	22	34	32	39	35	18	29	16	22	35	26	20	21	38	20
***V. antiquarius***																		
1‐2 (7‐a)	6	30	28	27	27	28	30	28	37	39	20	25	26	15	15	17	34	25
11‐4 (1)	6	32	30	29	29	30	29	29	39	40	21	24	30	20	19	19	33	26
***V. fujianensis***																		
1‐1 (7)	6	26	26	22	32	31	36	32	21	29	18	17	33	29	19	19	35	19
12‐2 (3‐a)	12	25	27	25	34	33	42	39	23	31	17	24	39	34	21	21	39	24

Abbreviations: AMP, Ampicillin; ATM, Aztreonam; AZM, Azithromycin; CAZ, Ceftazidime; CIP, Ciprofloxacin; CN, Gentamicin; CTX, Cefotaxime; DO, Doxycycline; E, Erythromycin; FFC, Florfenicol; IPM, Imipenem; MEL, Mecillinam; MEM, Meropenem; OA, Oxolinic acid; SXT, Sulfamethoxazole/Trimethoprim; TE, Tetracycline; TOB, Tobramycin; W, Trimethoprim.

**Table A2:** Assembly statistics for whole‐genome sequenced *Vibrio* spp

Isolate	Species	Accession no.	Coverage	Total length	No. Contigs	GC (%)	N50	CDSs (Total)
B4‐6	*V. anguillarum*	VHSL00000000	54.8X	3,901,483	69	44.51	300,519	3,560
B7	*V. anguillarum*	VHSN00000000	37.1X	3,954,657	40	44.64	283,309	3,541
B1‐2	*V. anguillarum*	VHSK00000000	52.4X	3,987,976	45	44.54	242,254	3,677
B4‐12	*V. anguillarum*	VHSM00000000	37.3X	3,965,239	48	44.65	283,309	3,555
B8‐1	*V. anguillarum*	VHSO00000000	60.9X	3,954,672	41	44.64	283,302	3,540
A8‐1	*V. metschnikovii*	VHTC00000000	30.2X	3,761,458	133	44.31	98,018	3,543
A11	*V. metschnikovii*	VHSI00000000	55.8X	3,803,682	81	44.14	234,690	3,464
2‐1 (7)	*V. alginolyticus*	VHSR00000000	49.2X	5,228,382	24	44.61	1,096,303	4,869
2‐2 (2)	*V. alginolyticus*	VHSS00000000	38.1X	5,175,814	36	44.63	604,436	4,806
2‐2 (7)	*V. alginolyticus*	VHST00000000	31.1X	5,176,990	39	44.58	501,798	4,812
2‐2 (9)	V. alginolyticus	VHSV00000000	5.61X	5,444,598	416	44.53	112,490	5,262
7‐5 (1‐a)	*V. alginolyticus*	VHSX00000000	67.5X	5,176,459	36	44.63	1,221,862	4,806
3‐2(1)	*V. alginolyticus*	VHSW00000000	79.8X	4,110,525	66	44.37	423,004	3,731
2‐2(8)	*V. alginolyticus*	VHSU00000000	38.1X	3,980,405	55	44.53	368,657	3,677
1‐2 (7‐a)	*V. antiquarius*	VHSQ00000000	57.4X	5,204,341	32	44.77	494,432	4,786
11‐4 (1)	*V. antiquarius*	VHSY00000000	31.7X	5,251,311	67	44.79	442,253	4,937
1‐1 (7)	*V. fujianensis*	VHSP00000000	67.8X	3,651,083	37	43.48	834,663	3,560
12‐2 (3‐a)	*V. fujianensis*	VMQP00000000	40.8X	3,650,909	41	43.38	834,684	3,248

Abbreviation: CDSs, Coding sequences.
